# Endolymphatic sac surgery: past and present controversies

**DOI:** 10.3389/fneur.2025.1635186

**Published:** 2025-07-28

**Authors:** W. P. R. Gibson

**Affiliations:** Department of Surgery, The University of Sydney, Sydney, NSW, Australia

**Keywords:** vertigo, Meniere’s disease, endolymph, endolymphatic sac, surgery

## Abstract

The history of endolymphatic sac surgery is presented. The original concept of passive drainage is challenged as the physiology of the endolymphatic sac is gradually elucidated. Surgery causes damage to the delicate cells with the concept of endolymphatic sac excision and endolymphatic duct clipping discussed. The author presents a hypothesis of how vertigo attacks occur.

## Introduction

Meniere’s disease (MD) is characterized by episodic attacks of vertigo associated with a fluctuating progressive hearing loss, tinnitus and often a feeling of fullness in the affected ear. Initially the vertigo attacks cause the most distress but as the disease progresses the vertigo becomes less severe and finally ceases when the hearing has declined (1). Many different surgical treatments have been suggested to arrest the attacks of vertigo (2). One treatment was endolymphatic sac (ELS) surgery which has remained highly controversial.

### Some anatomical and physiological details

**Figure 1:**
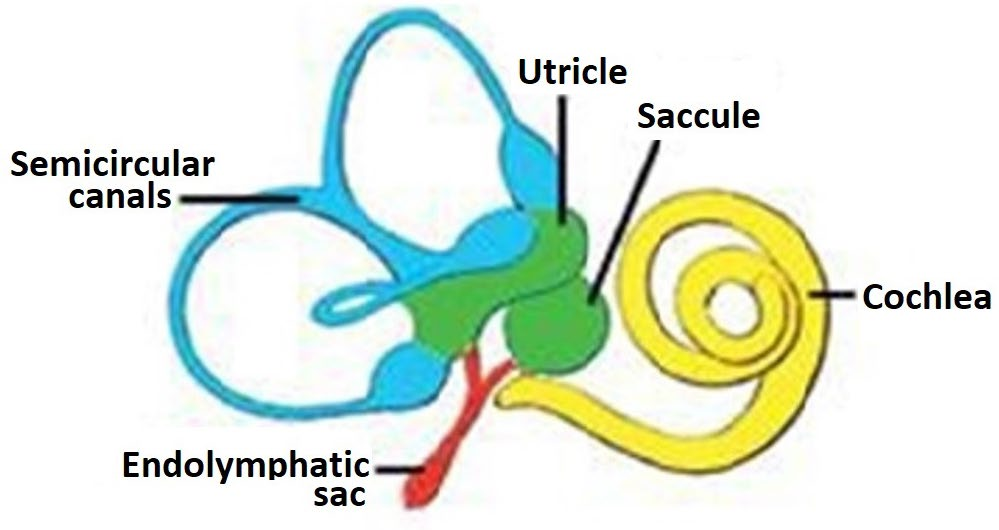
A simple diagram of the components of the inner ear. Yellow-pars inferior, green and blue- pars superior, red- endolymphatic duct and sac.

The endolymphatic sac (ELS) was first described in 1760 by Domenico Cotugno when he was only 24 years of age (3). A diagram shows the basic parts of the inner ear (Figure 1). The human ELS was once believed to be a functionless vestige of our phylogenetic past. To some otologists the ELS was merely the umbilicus of the inner ear. How wrong they were as studies, especially those in Uppsala have shown the intricate structure and functions of the ELS responsible for regulating endolymph volume and pressure, immune responses, and waste product removal (4–6).

**Figure 2:**
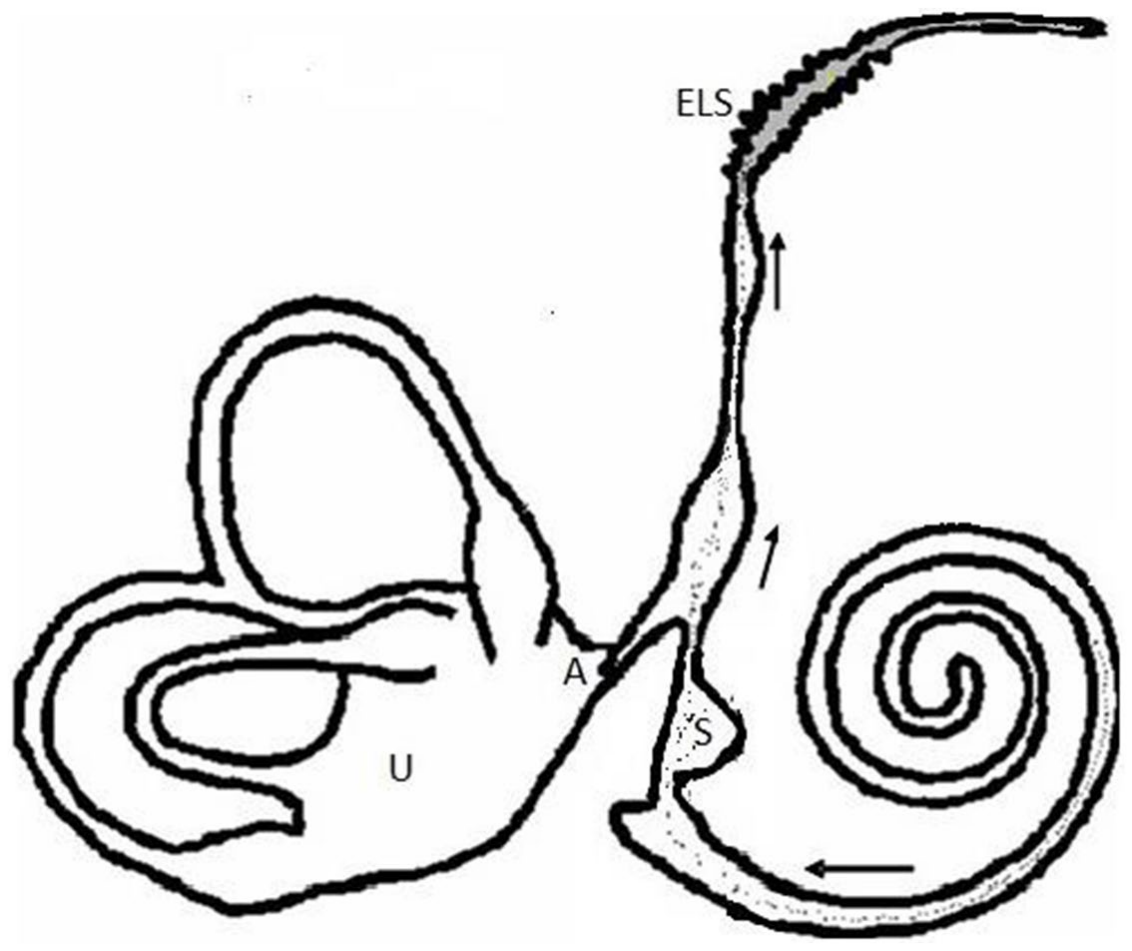
A diagram of longitudinal drainage (A—The valve of Bast (Utricular valve) which prevents drainage from the utricle during flow from the cochlea. S—saccule, U—Utricle, ELS—Endolymphatic sac).

The human ELS differs from that of a guinea pig. In the guinea pig there is a lumen whereas the human ELS has a complex series of folds with little evidence of a lumen (7). It still is a matter of conjecture how excess endolymph in the inner ear reaches the ELS. Initially it was believed to be a rise in pressure of the endolymph within the cochlea that sent the fluid towards to a passive ELS. Alternatively, the ELS may attract endolymph by secreting glycoprotein, a powerful hydrophilic substance, and then removing the waterlogged glycoprotein by macrophages and enzymatic activity (8) (see Figure 2).

### Endolymphatic hydrops (EH)

In1938, Hallpike and Cairns demonstrated distension of the cochlear duct in the temporal bone of a MD patient who died after vestibular nerve section (9). In the same year, Yamakawa published a similar finding independently (10). Reissner’s membrane becomes displaced by excess endolymph, known as EH. Subsequently, many studies have confirmed the presence both histologically (11) and by using magnetic resonance imaging (MRI) (12). The longitudinal flow of endolymph to the ELS is not continuous but has been shown to only occur when there is an excess of endolymph (13). Endolymph volume is usually maintained by radial flow.

### The history of endolymphatic sac surgery

The first ES surgery was performed by Portmann (14). He exposed the ES through the mastoid bone and made an incision into its lumen. At the time it was thought that there was increased pressure within the inner ear and this operation would alleviate the pressure and prevent the attacks of vertigo.

In 1963, Schucknect devised a ‘rupture’ theory based on his own histological studies (15). He postulated that a sudden increase of endolymph, could rupture Reissner’s membrane. This would mix the endolymph with the perilymph around the around the afferent vestibular nerve and the increase in potassium would cause a temporary paralysis causing the vertigo until the chemical composition was restored and Reissner’s membrane healed.

In 1967, Kimura showed that blocking the endolymphatic duct (ED) in guinea pigs causedEH (16). After blocking the ED on cats, only mild EH develops after many months (17), and, in monkeys, EH is even milder and takes longer to develop (18). During the 1960s and 1970s, ELS surgery became a popular treatment to prevent distressing attacks of vertigo. The surgery was designed to enhance drainage of endolymph to the ELS. Various surgical techniques were performed to enhance drainage. House performed a shunt into the subarachnoid space claiming a 83% success in preventing the attacks of vertigo (19). However, a subsequent study showed there was no advantage of the subarachnoid shunt compared with a mastoid shunt and the sub arachnoid shunt was abandoned (20). Others placed sialastic within the ELS (21) or placed a T tube into the lumen (22). In 1978, Arenberg added a one-way valve to the shunt tubing (23). Some surgeons found that it was sufficient to merely expose the ELS in the mastoid and decompress it and a study showed that merely decompressing the ELS was as effective as opening it (24). In 1996, Gibson removed the extraosseous portion of the ELS as he believed that the ELS was responsible in some way for initiating the attacks of vertigo (25). In 2015, Saliba and coworkers described the technique of clipping the endolymphatic duct (26), and this has also been described by Schenck (27). Interestingly all the above methods of ELS surgery, both non-destructive and destructive, provided 70–80% relief from the attacks of vertigo.

### The major study in 1981

In 1981, Thomsen and his colleagues published a game-changing study (28). They presented a double-blind controlled trial which showed no significant difference between ELS shunt surgery and a merely opening the mastoid bone (placebo group). The trial involved 15 patients in each group. The greatest difference was between the preoperative and postoperative scores, as both groups improved significantly. They concluded that that the 70% improvement in both groups was most likely caused by a placebo effect. Attempts were made to discredit this study (29, 30), but the patients were followed up for some years and still no significant differences were observed (31, 32). A second controlled study was performed by the Thomsen comparing ELS surgery with grommet insertion which showed no significant differences between the groups (33).

After the study by Thomsen et al., there was a decline in the popularity of ELS surgery.

### Further relevant research after 1981

There has been increasing skepticism regarding the rupture theory (15). It was a concern that a rupture within the cochlear duct would alter the perilymph composition in the pars superior without having a profound effect on audition. In humans, recordings have been made of the pure tone audiogram during attacks which do not show significant change in the hearing threshold during the attacks (34). An animal study by Brown and co-workers described an experiment when artificial endolymph was introduced into the cochlea duct of the guinea pig causing an increase in the summating potential (SP) and a decrease in the action potential (AP) threshold (35). Then, suddenly the SP decreased back to normal levels and the AP improved. It was initially thought that this was due to a rupture, but detailed micro-CT examination failed to reveal any ruptures of Reissner’s membrane, and there was no leakage of fluorescein into the perilymphatic compartment. They surmised that the sudden change was due to the opening of the utricular valve of Bast sending the excess endolymph into the utricle.

Alternative theories for the attacks of vertigo have been suggested. The distension theory suggests that a change in endolymph pressure stimulates the vestibular hair cells (36). Another theory was that leakage of potassium into the perilymph occurs without a rupture (37). Gibson and Arenberg postulated that the ELS reacts hyperactively inducing rapid drainage of endolymph (38). The theory was modified to suggest that during periods of rapid drainage the endolymphatic sinus becomes overfull and a reflux of cochlear endolymph into the utricle stimulates the vestibular haircells (39). A similar theory has recently been suggested by Li et al. (40).

Based on the concept that the ELS has a role in initiating the attacks of vertigo, Gibson proposed removing the extraosseous portion of the endolymphatic sac in 1996 (25). Favorable results were reported in 1999 (41) and 2000 (42). In contrast, Welling and co-workers found no significant differences between a small group of 10 patients after ELS excision and 10 patients after ELS shunts (43).

More recently, Saliba et al. (26) and Schenck et al. (27) have reported favorable results after blocking the endolymphatic duct. The surgery involves exposing the extraosseous portion of the ELS by the trans-mastoid route. The entrance of the ELS into the operculum is identified and clipped using two titanium clips (44). Gibson was concerned that the ELS might transmit a hormone to the cochlea to produce the excess endolymph and decided to remove as much of the ELS as possible. However, Li and his co-workers have shown that the ELS produces endolymph (40), so perhaps this explains why clipping the duct is effective. A study by Peng and colleagues did not show any reduction of endolymphatic hydrops after endolymphatic sac blockage or other methods of ELS surgery (45).

The ELS is a delicate structure, and it is likely that any surgery to insert a tube or sialastic into the intricate folds of lumen causes damage. Perhaps even exposing and decompressing the ELS causes damage. The fact that removal of the ELS or clipping of the duct does not worsen the vertigo strongly disproves the concept that ELS surgery promotes drainage.

### A possible hypothesis

It is suggested that the ELS acts to defend the inner ear. The defense of the hair cells and supporting cells is vital, as these cannot regenerate. Thus, a mechanism exists to transport viruses, bacterial remnants, dead cells, or other noxious agents to the ELS, which is immunologically competent, and can destroy the viruses and remove noxious agents off site and away from hair cell harm.

When the ELS senses a threat to the inner ear, it produces excess endolymph and then removes the excess endolymph by longitudinal flow, removing the noxious agents into the ELS.

The attacks of vertigo during Meniere’s disease are due to faulty longitudinal drainage. The common factor is that the vestibular aqueduct is narrow and longitudinal flow through the endolymphatic duct is impeded (46). Further flow restrictions may be due to genetic causes (47), otoliths narrowing the duct (48), a spirochete (49), a tumor of the ELS (50), etc. The restricted flow of excess endolymph towards the ELS during a period of rapid drainage causes an overflow of cochlear endolymph into the utricle stimulating the vestibular hair cells and causing an attack of vertigo (39).

One of the initial causes of Meniere’s disease could be the result of a viral labyrinthitis. The response to the inflammatory event causes increased endolymph production and then activates drainage to the ELS to remove the inflammatory debris.

A further concept is that there is a trigger volume of endolymph that can activate the longitudinal flow mechanism. Small increases of endolymph may be due to factors such as salt loading, infections, stress causing vasopressin release, etc. There is therefore a cluster of attacks of vertigo until the volume of endolymph decreases sufficiently to prevent further attacks of vertigo and a remission occurs.

It is hypothesized that the ELS, as an immune organ, has a memory. If the ELS realizes that another assault on the inner ear is prevalent, it can activate a mechanism that increases endolymph volume to initiate longitudinal flow.

One possible theory is that there is an initial viral cause, and antibodies are created in the ELS. If a similar virus that caused the initial viral labyrinthitis enters the body and circulates in the blood but cannot be eliminated, then the antigens will reach the ELS and cause an immune reaction to defend the inner ear. There may also be auto-antibodies which target ELS antigens. One proposed mechanism involves molecular mimicry, where viral or bacterial antigens share structural similarities with the memorized ELS antigens leading to a cross-reactive immune response (51).

After repeated drainage episodes, the glycoprotein production begins to cripple the ELS causing fibrosis. Eventually the ELS becomes functionless, and the attacks of vertigo cease but there is widespread EH causing loss of hair cell function.

## Conclusion

Meniere’s disease is probably not a single disease but several different etiologies which can produce the syndrome of attacks of vertigo, hearing loss, tinnitus and aural fullness. ELS surgery remains a controversial treatment. Patients become desperate when suffering attacks of vertigo and may seek a surgical solution. At least 70 % will get relief after ELS surgery whether this has truly altered the pathophysiology or whether it was merely a placebo response. Removal of the ELS or clipping the duct probably hastens the progression of the disease to its ‘burnt-out stage’. An advantage in comparison with intratympanic gentamicin therapy, labyrinthectomy or vestibular nerve section, is that there is rarely any loss of vestibular function (52). Perhaps there is a role especially for elderly patients.
